# Knowledge, attitude and occupational risks to hepatitis B infection among health workers in Gulu Regional Referral Hospital, Northern Uganda: a cross-sectional study design

**DOI:** 10.11604/pamj.2021.39.138.23724

**Published:** 2021-06-17

**Authors:** Morris Ojara, Gloria Owomugisha, Isaac Staron Kibunga, Lucy Grace Asio, Ibrahim Bwaga, Thomas Nabugere, Richard Martin Tuwayenga, Eric Nzirakaindi Ikoona, David Lagoro Kitara

**Affiliations:** 1 Department of Surgery, Faculty of Medicine, Gulu University, P .0. Box 166, Gulu, Uganda,; 2 ICAP at Columbia University, Freetown, Sierra Leone,; 3 Department of Global Health and Populations, Harvard T.H. Chan School of Public health, Harvard University, Boston, Massachusetts, United States of America

**Keywords:** Hepatitis B, Gulu Regional Hospital, knowledge, attitude, health workers, occupational risks, Uganda

## Abstract

**Introduction:**

hepatitis B virus (HBV) is one of the commonest causes of acute and chronic liver diseases worldwide. HBV can be transmitted by exposure to infected blood and human secretions through sharp injuries and splashes. Health workers are among the most high-risk groups because they regularly interact with patients. A seroprevalence survey conducted in Uganda in 2014 found a higher prevalence of HBV in Gulu Municipality compared to the rest of Uganda.

**Methods:**

a cross-sectional study was conducted among health workers in Gulu Regional Hospital. A stratified random sampling was used. Knowledge ratings and Likert scale were used to score knowledge, attitudes and risks of HBV infections in a qualitative assessment. Ethical approval was obtained and SPSS was used for data analysis. A p-value less than 0.05 was considered significant.

**Results:**

one hundred and twenty-six (126) respondents participated; 65 (51.6%) were male, 80 (63.5%) were aged 20-29 years, 74 (58.7%) were not married, 86 (68.3%) had a work experience of 0-9 years, 64 (50.8%) had good knowledge, 90(71.4%) had positive attitude, 114 (90.5%) had high to very high pre-exposure risks, and 75 (59.5%) had moderate to high exposure and post-exposure risks. There was no significant difference in knowledge (*X*^2^= 13.895; p = 0.178) and work experience (*X*^2^= 21.196; p = 0.097) among the health workers.

**Conclusion:**

there is a high pre-exposure, exposure and post-exposure risks of HBV infection among health workers in Gulu Hospital. There is need to augment awareness on HBV infection and design strategies to strengthen and implement infection control measures including HBV vaccination among health workers.

## Introduction

Hepatitis B virus (HBV) is one of the commonest causes of acute and chronic liver diseases worldwide [1]. Uganda is among the most highly endemic countries in the sub-Saharan Africa with more than 1.4 million adults chronically infected [1]. The prevalence of HBV surface antigen (HBsAg), a biomarker of chronic HBV infection, ranged from 6% to 15% among blood donors when HBV screening was introduced in some selected populations in Uganda [1]. In 2005, one in ten adults in Uganda had HBV surface antigen with an estimated 1.4 million people living with active disease compared to 900,000 Ugandans living with human immune deficiency virus (HIV) and acquired immune-deficiency syndrome (AIDS) [2]. HBV when present in serum, is usually found in large quantities (108 to 1010 virions/ml) and can be detected in semen, saliva, cervical secretions and leukocytes [3]. The major route of HBV transmission in sub-Saharan Africa is mainly by horizontal route (that is transmission unrelated to the recognized methods such as sexual intercourse, perinatal and parenteral exposures) [4]. HBV is highly resilient; resistant to breakdown, and thus can survive outside the body [4]. HBV can easily be transmitted through contacts with infected body fluids [4], putting health workers who are exposed to infected body fluids through Needle Stick Injuries (NSIs) and splash incidents during body contacts with patients, occupationally at high risk to HBV infection [5].

A study conducted in Mulago National Referral Hospital (MNRH) in Uganda in 2010 found the rate of NSI as 4.2 per person-year with 300/526 (57%) of nurses and midwives interviewed acknowledging having experienced at least one NSI in the previous year and only 95/526 (18%) acknowledging not having experienced NSI in their entire career [5]. A study conducted on NSIs in the sub-Saharan Africa in 2005 found that the strongest predictor of NSIs among health workers was lack of training, long work hours, poor work conditions, work fatigue, poor work habits, lack of knowledge, and lack of experience [6]. HBV infection is a significant public health problem because it may lead to chronic infection, resulting in liver cirrhosis, liver cancer, liver failure, and death [7]. Several extra-hepatic lesions also occur due to the deposition of immune complexes in different organs, especially in the kidneys [8]. Also, a person with chronic HBV infection serves as the main reservoir for its continued transmission among contacts [9]. There are an estimated 360 million HBV chronic infections worldwide, with most of them living in South East Asia and sub-Saharan Africa [10, 11]. Approximately 50 million chronic carriers of HBV are in Africa, with an estimated mortality risk of 25% [12]. A study conducted in a rural healthcare setting in northern India showed that health workers were at risks of infection with blood-borne viruses such as HBV, hepatitis C virus (HCV), and HIV through contacts with blood and other body fluids during the course of their work [13]. A recent cross-sectional population-based study conducted in Gulu Municipality in Northern Uganda found that there was a very high prevalence of HBV infection of 17.6% with a lifetime exposure of 72.4% in the general population [14]. The same study also found HBV infection rates and lifetime exposures in children as 21.9% and 48% respectively [14]. This study was conducted among health workers in Gulu Regional Referral Hospital to assess knowledge, attitude and occupational risks to hepatitis B virus infection.

## Methods

**Study design:** a cross-sectional study was conducted among health workers in Northern Uganda.

**Study site:** the study was conducted in Gulu Hospital, a Regional Referral and Public University Teaching Hospital located in the center of Gulu Municipality, approximately 343km (213 miles) by road north of Kampala, the capital city of Uganda. It is a referral hospital for Amuru, Gulu, Kitgum, Agago, Omoro, Nwoya, Lamwo, and Pader districts and serves as one of the teaching hospitals for Gulu University Medical School, Gulu clinical officers´ training school and Gulu nurses´ training school. It has a capacity of approximately 389 beds with several Departments: Surgery, accident and emergency, internal medicine, paediatrics and child health, reproductive health, operating theatre, laboratory, radiology, pathology, community health, psychiatry and mental health, adolescent health, orthopaedics and rehabilitation, ophthalmology and general out-patient departments. It has specialized clinics such as; nutrition, TB, sickle cell disease, diabetes, HIV, dermatology, cardiology, hypertension, dental, and Ear, Nose and Throat. It employs approximately 361 health workers and facilitates training for over 300 students in clinical years.

**Target population:** our target population was health workers in Gulu Regional Referral Hospital (GRRH).

**Study population:** health workers in GRRH in clinical departments.

### Selection criteria

**Inclusion criteria:** employees of GRRH, medical students, nursing and clinical officers´ students in clinical rotations.

**Exclusion criteria:** health workers who had worked in GRRH for less than 3 months and employees who were not in the clinical departments.

**Sample size calculation:** sample size was determined using Kish, Leslie formula (1965)

$$N=\frac{Z^{2}pq}{d^{2}}$$

Where, N = estimated sample size; Z=level of significance which is 1.96, (95% Confidence Interval).

p = the estimated proportion of health workers exposed to Hepatitis B virus while at work which is 0.09 [15].

q = (1-p) = (1- 0.09)

d = the precision of the estimate expected which is 0.05.

Therefore: N = (1.96)^2^x 0.09 x (1-0.09) / (0.05)^2^= 126

We took into consideration non-response rate of approximately 10% and rounded our sample size to 140.

### Sampling procedure

To obtain the required sample size, we got the employee list from the human resource officer of GRRH and for clinical year students from their respective training institutions. We calculated the total study population by adding the numbers of health workers in their strata as; Doctors (specialists, medical officers, and intern doctors); students (medical students in third, fourth, and fifth years, nursing students, and clinical officers´ students), laboratory technicians, nurses, clinical officers and others (theatre assistants, nursing assistants, and midwives). That gave a total eligible sample population of 352 health workers in the clinical departments. The sample size per stratum (contribution of each stratum to the overall sample size) was calculated using the formula below;

$$N=\frac{\text{number of health workers in a category}}{\text{Total number of health workers}}\times \text{sample size}$$

Thus as per the records, the eligible population were as follows: doctors (20), nurses (135), clinical officers (38), laboratory technicians (9), and students (150). We calculated the sample size per stratum; doctors (8), nurses (54), clinical officers (15), laboratory technicians (4), and students (60). However, due to un-matching number of staff in the hospital record with physical counts, the actual sample size realized for each stratum selected were; doctors (5), nurses (37), clinical officers (23), laboratory technicians (7), students (49), and others (midwives, nursing and theatre assistants) (5). At this stage, the selection of specific cadre participant in each stratum was conducted randomly, whereby the questionnaire was administered to the health worker who consented and was available at the work station in the specific department at the time of this study, until we achieved the required sample size.

### Data collection procedures

**Research instrument:** the main instrument for data collection was a questionnaire designed with open and closed-ended questions, including the socio-demographic characteristics, knowledge, attitude, pre-exposure, exposure, and post-exposure risks of participants.

**Data collection technique:** data collection was conducted using interviewer-administered questionnaire

**Determination of knowledge of respondents to hepatitis B virus:** to determine the level of knowledge of respondents, a modified knowledge rating by the research team was used. The questionnaire had eleven questions on general knowledge on HBV and its complications. For each correct answer got, the respondent earned a score of 1 point (Yes as 1 and No as 0). The sum of all these answers per respondent constituted the knowledge rating of that individual. The potential range of knowledge scores for each respondent varied between 0 and 11. The aggregated score for each respondent was then categorized as very poor knowledge (scores of< 2 or < 18%), poor (scores of 3 to 5 or 27.3-45.5%), good (scores of 6 to 8 or 54.5-72.7%), and very good (scores of 9 to 11 or 81.8-100%) (Annex 1).

### Determination of attitudes of respondents to hepatitis B virus

A modified Likert scale was used to determine the attitude of respondents to HBV. There were seven statements to assess respondents´ attitudes towards HBV in the questionnaire (these were general precautionary measures and actions that should be undertaken to prevent the potential risks of acquiring HBV while on duty at Gulu Regional Hospital). All these questions had a 5-point Likert scale [16, 17], with each of them structured negatively and the best possible answer as strongly disagree. Thus, the possible responses to each question were arranged as follows; (0 for strongly agree, 1 for agree, 2 for indifferent, 3 for disagree, and 4 for strongly disagree). The maximum possible scores for attitudes of each respondent ranged between 0 and 28. The aggregated scores for each respondent was categorized as very negative attitude (scores of < 7 or < 25%), negative (scores of 8-14 or 28.6-59%), neutral (scores of 15-21 or 53.6-75%), and positive (scores of 22-28 or 78.6-100%) (Annex 1).

**Occupational risks of respondents to hepatitis B virus**

### Determination of pre-exposure risks of respondents to HBV

The questionnaire had sixteen questions to assess pre-exposure risks to HBV infection (This assessed the general precautionary measures respondents should have undertaken or should undertake to prevent HBV infection during work at Gulu Regional Hospital). These 16 questions on HBV pre-exposure risks had the highest score of four (with always as 0, most of the times as 1, sometimes as 2, rarely as 3, and never as 4); three questions with the highest score of two (Yes as 0, No as 1 and I do not know as 2), and two questions with the highest score of one (Yes as 0 and No as 1) in the questionnaire. Accordingly, the potential range of aggregated scores for each respondent ranged from 0 and 72. In the pre-exposure category, the higher the scores, the higher the risks, and vice versa. Hence, the most minimal possible score for each respondent was 0 and a maximum of 72. Subsequently, the pre-exposure risks of each respondent obtained from the aggregated score were categorized as follows; very high (scores of 58-72 or 80.6-100%), high (scores of 44-57 or 61.1-79.2%), moderate (scores of 29-43 or 40.3-59.7%), low (scores of 15-28 or 20.8-38.9%), and very low (scores of 0-15 or 0-20.8%) (Annex 1).

### Determination of exposure and post-exposure risks of respondents to HBV

In determining the exposure and post-exposure risks, joint assessment of the two risks were conducted and the scores obtained were directly proportional to the risks of getting infected with hepatitis B virus. Questions on exposure and post-exposure risks assessed the actions taken or actions that should have been taken by each respondent so as to avoid acquiring hepatitis B virus when he/she got exposed during work at Gulu Regional Hospital. There were thirteen questions on exposure risks of respondents and the highest score for each question was one (Yes as 1 and No as 0). Post-exposure risks had five questions with the highest score for each question being one (Yes as 0 and No as 1). As a result, the minimum possible aggregated score (exposure and post-exposure) for each respondent was 0 and maximum 18. The aggregated scores for combined exposure and post-exposure risks per respondent were categorized as; very low; defined as scores of, 0-5 (0-27.8%), low as 6-8 (33.3-44.4%), moderate as 9-12 (50-66.7%), high as 13-15 (72.2-83.3%), and very high as 16-18 (88.9-100%) (Annex 1).

### Data quality control

Questions in the questionnaire for data collection were constructed, pilot tested and pre-tested before use. This was to ensure internal validity and reliability of the information obtained. The pre-test was conducted at St. Mary´s Hospital, Lacor since it had similar characteristics of health workers, close proximity and same catchment population to Gulu Regional Referral Hospital where the study was conducted. Questions were aligned in sequence to avoid any loss of information during interviews. The completed questionnaires were cross-checked to ensure completeness before moving to the next interview. The questions had an internal validity Cronbach´s alpha coefficient of more than 0.74.

### Data management and analysis

Data was entered and analyzed using SPSS version 16.0 and Epi Info version 7, and presented in tables and figures. A bivariate analysis to assess association between variables were conducted using Chi square tests and a p-value less than 0.05 was considered statistically significant.

### Ethical approval

This study was approved by the Gulu University Institutional Review Committee (GUIREC) and the management of Gulu Regional Referral Hospital. Informed consent was obtained from each study participant. De-identification of participants on the questionnaire ensured confidentiality of information, and the principle of autonomy and respect of respondents was maintained throughout the study.

## Results

This study was conducted among health workers of Gulu Regional Referral Hospital in 2013. In the 2014 population-based seroprevalence survey, Gulu Municipality had a higher prevalence of hepatitis B virus compared to other parts of Uganda. Table 1 represents the socio-demographic characteristics of respondents where the majority were in the young age-group, not married, males, nurses and had relatively short work experience of less than ten years.

**Table 1:** socio-demographic characteristics of respondents in Gulu Regional Referral Hospital

Variables	Frequency (n=126)	Percent (%)
**Age (years)**		
20-29	80	63.5
30-39	21	16.7
40-49	7	5.5
50-59	18	14.3
Total	126	100.0
**Sex**		
Male	65	51.6
Female	61	48.4
Total	126	100.0
**Marital status**		
Married	44	34.9
Single	74	58.7
Divorced	1	0.8
Others	7	5.6
Total	126	100.0
**Job catergories**		
Doctors	5	3.9
Clinical Officers	23	18.3
Nurses	37	29.4
Laboratory Technicians	7	5.6
Students	49	38.9
Others (Midwives, Nursing and theatre assistants)	5	3.9
Total	126	100.0
**Work experience (years)**		
0-9	86	68.3
10-19	21	16.7
20-29	9	7.1
30-39	10	7.9
Total	126	100.0

Table 1 summarizes the socio-demographic characteristics of respondents; 126(100%) were interviewed; age ranged from 20 to 59 years with a mean of 31.3(SD+11.1) years. The average work experience was 8.2(SD+9.5) years and this ranged from less than 1 to 36 years. The majority were 20-29 years old 80(63.5%); males 65(51.6%); single 74(58.7%); students 49(38.9%) and had a work experience of 0-9 years 86(68.3%).

### Knowledge of respondents on hepatitis B virus

In Table 2, the majority of health workers had good knowledge scores and grades on hepatitis B virus. Table 3 shows that while a higher proportion of laboratory technicians, clinical officers and nurses had better knowledge on HBV compared to the other health workers, this difference was not statistically significant. Similarly in Table 4, though a higher proportion of health workers with longer work experience (>10 years) had better knowledge, there was no significant difference in the study population.

**Table 2:** knowledge scores and grades of respondents on Hepatitis B virus

Knowledge scores	Frequency (n=126)	Percentage (%)
<2	0	0.0
3-5	61	48.4
6-8	64	50.8
9-11	1	0.8
Total	126	100.0
**Knowledge grades**		
Very poor	0	0.0
Poor	61	48.4
Good	64	50.8
Very good	1	0.8
Total	126	100.0

Table 2 shows the actual knowledge scores ranged from 3 to 9 with a mean 5.54 (SD+1.30), a median 6 and mode 6. The majority of respondents 64/126 (50.8%) had good knowledge; 61/126 (48.4%) had poor knowledge, and only 1/126 (0.8%) had very good knowledge.

**Table 3:** knowledge grades and job category of respondents to Hepatitis B virus

Job catergories								
**Knowledge grades**	**Laboratory Technician**	**Doctors**	**Clinical officers**	**Nurses**	**Students**	**Others**	**Total**	**Chi Square test**
Poor	2	5	7	16	28	3	61	χ2=13.895; p=0.178
Good	5	0	16	20	21	2	64	
Very good	0	0	0	1	0	0	1	
Total	7	5	23	37	49	5	126	

Table 3 shows a cross-tabulation between knowledge grades and job categories. A higher proportion of Laboratory technicians, clinical officers and nurses had good knowledge compared to the other categories. Most notably all the 5 doctors interviewed had poor knowledge, however there was no statistically significant difference in knowledge among the health workers (χ2=13.895; p=0.178).

**Table 4:** knowledge grades and work experience of respondents to hepatitis B virus

Work Experience (years)							
**Knowledge grades**	1-4	5-9	10-19	20-29	30-39	Total	Chi Square test
Poor	39	9	8	3	2	61	χ2=21.196; p=0.097
Good	31	7	13	5	8	64	
Very good	0	0	0	1	0	1	
Total	70	16	21	9	10	126	


Tables 4 shows a cross-tabulation between knowledge grades and work experience (years). A higher proportion of health workers with longer work experience (>10 years) had good knowledge compared to those with shorter work experience (<10 years). However, the knowledge difference between the groups with varying work experience was not statistically significant (χ2=21.196; p=0.097).

### Views of respondents on routes of transmission of hepatitis B virus

**Figure 1:**
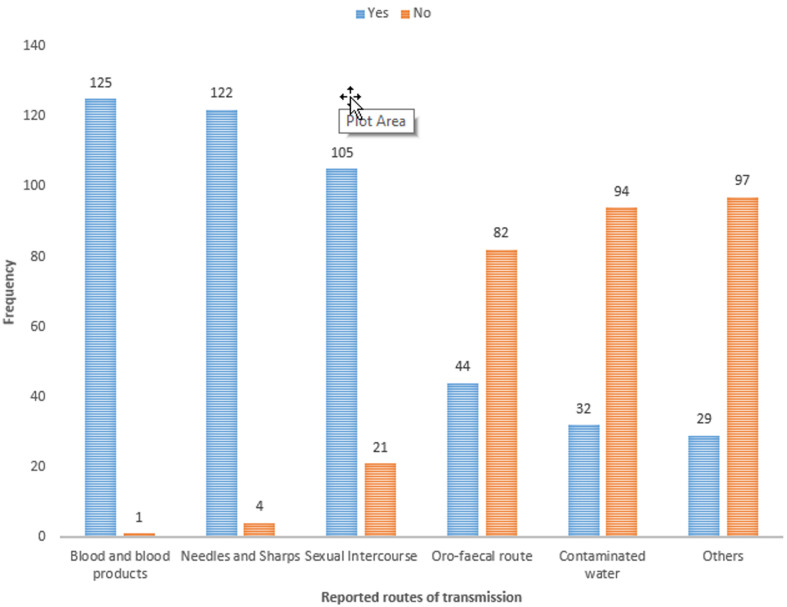
respondents’ views on the routes of transmission of hepatitis B virus

Figure 1 is a bar graph presenting the views of respondents on the routes of transmission of hepatitis B virus as; blood and its products, needles and sharp injuries, sexual intercourse, oro-faecal route, contaminated water and vertical transmission among many others in the descending order.

### Findings on prevention methods to HBV among respondents

All respondents 126 (100%) knew that hepatitis B vaccine was available, 122 (96.8%) knew the correct dosing but only 56 (44.4%) knew the expected interval between the last dose and the preceding dosage.

### Attitudes of respondents to hepatitis B virus

In Table 5, the majority of health workers 90 (71.4%) had positive attitudes. Table 6 shows the majority of the health workers had high 74 (58.7%) and very high 40 (31.8%) pre-exposure risks. As for the exposure and post-exposure risks, the majority had very low risks 44(34.9%), followed by moderate 43 (34.1%) and high 32 (25.4%) risks in that order.

**Table 5:** attitude scores and grades of respondents to Hepatitis B virus

Attitude scores of respondents	Frequency (n=126)	Percentage (%)
<7	0	0.0
18-14	3	2.4
15-21	33	26.2
22-28	90	71.4
Total	126	100.0
**Attitude grades of respondents**		
Very negative	0	0.0
Negative	3	2.4
Neutral	33	26.2
Positive	90	71.4
Total	126	100.0

Table 5 shows the attitude scores; it ranged from 11 to 28 with a mean score 23.07 (SD+3.84), a median 24.0 and mode 24. The majority of health workers in Gulu Regional Referral Hospital had positive attitudes towards Hepatitis B virus 90 (71.4%).

**Table 6:** occupational risks of respondents to hepatitis B virus

Pre-exposure risk scores	Frequency (n=126)	Percentage (%)
0-15	0	0.0
16-28	1	0.8
29-43	11	8.7
44-57	74	58.7
58-72	40	31.8
Total	126	100.0
**Grades of pre-exposure risks**		
Very high	40	31.8
High	74	58.7
Moderate	11	8.7
Low	1	0.8
Very low	0	0.0
Total	126	100.0
**Exposure and post-exposure risk scores**		
0-5	44	34.9
6-8	7	5.6
9-12	43	34.1
13-15	32	25.4
16-18	0	0.0
Total	126	100.0
**Grades of exposure and post-exposure risks**		
Very low	44	34.9
Low	7	5.6
Moderate	43	34.1
High	32	25.4
Very high	0	0.0
Total	126	100.0

Table 6 shows the actual pre-exposure risk scores which ranged from 24 to 68 with the majority 74 (58.7%) with high and 40 (31.8%) very high risks. For the exposure and post-exposure category, the majority had very low risks 44 (34.9%), followed by moderate 43 (34.1%) and high 32 (25.4%) risks in that descending order respectively.

### Screening and vaccination reports on hepatitis B virus

More than half of respondents 73 (57.9%) were screened and vaccinated against HBV however, only 54 (42.9%) completed all the three dosages. The majority 88 (69.8%) were trained on how to handle and dispose infectious materials. However, the majority 93 (73.8%) had not seen posters demonstrating risks involved in improper handling of infectious materials.

### Summary on exposure and post-exposure events by respondents

**Figure 2:**
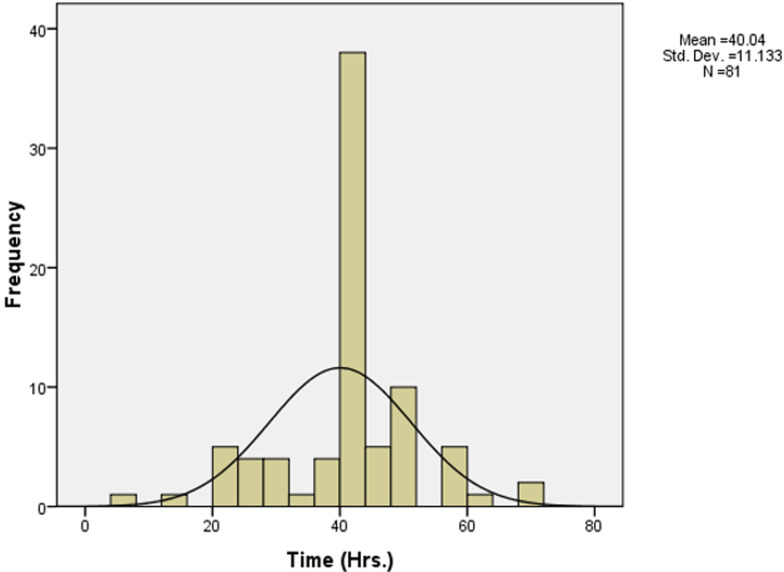
weekly work hours of respondents

Most respondents 82/126 (65.1%) reported having accidentally got into direct contact with body fluids of HBV patients in the last five years. Of the exposed, the majority, 61/82 (74.4%) was with blood, 19/82 (23.2%) with amniotic fluid, and 1/82 (1.2%) with cerebrospinal and pleural fluids. In these incidents, most 31/82 (37.8%) resulted from direct contacts with intact skin, 13/82 (15.9%) with broken skin, 14/82 (17.1%) through mucosal exposure of the mouth and eyes by splashing fluids, 19/82 (23.2%) from NSIs, 1/82 (1.2%) from a cut with a sharp object, and 3/82 (3.7%) from other mechanisms. The frequency of exposures among respondents was; once 13 (15.9%), twice 20 (24.4%), thrice 13 (15.9%), four times 1 (1.2%), and five or more times 32 (42.7%). Figure 2 is a histogram which shows the majority of respondents who got exposed to HBV had longer weekly average work hours.

### Attitudes of respondents to pre and post-exposure risks to hepatitis B

In determining the attitudes to HBV infection, the research team assessed responses of respondents on seven areas as follows: 1) Whether their work as health workers didn´t put them at risk to hepatitis B infection; 7(5.6%) strongly agreed, 12 (9.5%) agreed, 1 (0.8%) was indifferent, 30 (23.8%) disagreed and 76 (60.3%) strongly disagreed. 2) Whether there was no need to always wear gloves when conducting vene-puncture on a hepatitis B infected patient; 6 (4.8%) strongly agreed, 2 (1.6%) agreed, 8 (6.3%) disagreed, and 110(87.3%) strongly disagreed. 3) Whether they shouldn´t treat every patient as though they had a blood borne pathogen; 5 (4.0%) strongly agreed, 22 (17.5%) agreed, 7 (5.6%) were indifferent, 30 (23.8%) disagreed and the majority 62 (49.2%) strongly disagreed. 4) Whether an infected health workers couldn´t infect a patient with HBV; 3 (2.4%) strongly agreed, 3 (2.4%) agreed, 3 (2.4%) were indifferent, 37 (29.4%) disagreed and 80 (63.5%) strongly disagreed. 5) Whether they wouldn´t allow their relatives to be treated by a hepatitis B infected health worker; 25 (19.8%) strongly agreed, 10 (7.9%) agreed, 15 (11.9%) were indifferent, 40 (31.7%) disagreed and 36 (28.6%) strongly disagreed. 6) Whether hepatitis B vaccine was ineffective; 2 (1.6%) strongly agreed, 1 (0.8%) agreed, 5 (4.0%) were indifferent, 40 (31.7%) disagreed, and majority 78 (61.9%) strongly disagreed. 7) Whether they wouldn´t report needle stick injuries; 2 (1.6%) strongly agreed, 1 (0.8%) were indifferent, 22 (17.5%) disagreed, and majority 101 (80.2%) strongly disagreed.

**Figure 3:**
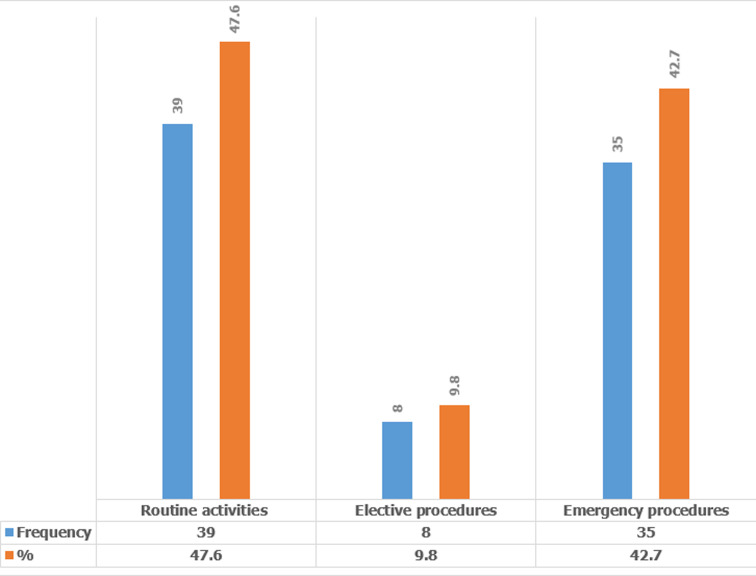
nature of procedures at the time of the exposure incidents

In summary, the questions on attitudes and pre-exposure risks, the majority of respondents disagreed and strongly disagreed with the seven questions in sequence as follows: 1^st^, 106 (84.1%); 2^nd^, 118 (93.6%); 3^rd^, 92 (73.0%); 4^th^, 117 (92.9%); 5^th^, 76 (60.3%); 6^th^, 118 (93.7%) and 7^th^, 123 (97.6%) respectively. These confirmed that most health workers had a positive attitude to pre-exposure risks to HBV infection. Figure 3 is a bar graph which describes the nature of procedures at the exposure incidents. The majority of the incident events were during routine procedures such as injections and institution of intravenous lines.

**Practices of respondents after exposure to HBV:** the majority of respondents 72/82 (87.8%) immediately washed the exposed parts with water and soap. Only 6/82 (7.3%) of the source patients were tested for HBsAg after the exposure incident. For health workers whose HBV status was unknown, 60/82 (73.2%) were not tested for either HBsAg or hepatitis B surface antibody (HBsAb), while 10/82 (12.2%) was tested for HBsAg, 1/82 (1.2%) for HBsAb, and 10/82 (12.2%) for both HBsAg and HBsAb.

### Occupational risks of respondents to hepatitis B

The majority of the respondents 73/126 (57.9%) were screened and vaccinated against HBV infection while 17/126 (13.5%) were only screened but not vaccinated. In comparison, 36/126 (28.6%) were neither screened nor vaccinated. Only 54/126 (42.9%) completed all three doses of HBV vaccines as per Ugandan Ministry of Health guidelines. 23/126 (18.3%) did not know that guidelines for HBV infection prevention and control were displayed on notices in Gulu hospital, and only 15/126(11.9%) had seen the guidelines. The majority 88(69.8%) were trained on handling and disposal of infectious materials. The majority 93/126 (73.8%) have never seen posters demonstrating risks involved in improper handling of infectious materials in Gulu hospital.

## Discussion

### Socio-demographic characteristics of respondents

The socio-demographic characteristics of respondents mirrored previous studies conducted in Northern Uganda [14, 18]. Similar to the current study, previous studies in Uganda found that the majority of health workers were generally young people, single (not married), and had short work experience (Table 1) [14, 18]. Like the current study, other researchers have found high prevalence of HBV increased in developing countries, especially where HBV prevention measures were not being practiced by health workers [19-21]. The high prevalence of HBV among health care workers is projected to create spheres of HBV infections in the population, mainly through accidental exposures to patients they treat. Long-term exposures to HBV for the relatively young health workers as we have seen in this study population may result into multitudes of complications to individual person with a possibility of poor quality of life and low quality-adjusted-life-years. This may have the potential of increasing absenteeism from work, job losses, poor income, socio-economic instability in families, early deaths and poor health care service delivery in health facilities if the health worker become sick due to HBV infection. In addition, poor health and income may promote the vicious cycle of poverty in families. Besides, this has implications on the health system´s resilience and sustainability especially in the human resource sector which is one of the major challenges faced by the health systems in Africa. It is hoped that with proper roll out of HBV vaccination, regular use of protective wears among health workers, prevention of horizontal and vertical transmission of HBV in the society, the incidence and prevalence of HBV in this community will improve.

### Knowledge of respondents on hepatitis B virus

Several reports show that better knowledge on HBV were more likely to provide protection to health workers for example; knowledge on HBV transmission as a nosocomial infection, routes of transmission (Figure 1), vaccination and occupational hazards [21, 22]. In the current study, most respondents were aware about HBV infection and its wide spread occurrence in this community which presents an opportunity for health managers to institute and reinforce HBV prevention measures, including HBV vaccination. Studies have demonstrated a positive relationship between better knowledge on HBV and the likelihood of the health workers adhering to HBV-related IPC measures [21-23]. Also, the majority were aware that NSIs and splash events were the commonest modes of transmission of HBV among health workers however, we argue that knowledge alone among health workers may not be sufficient to warrant protection to the virus but rather the attitudes and practices on prevention measures. Studies conducted outside sub-Saharan Africa however found insufficient knowledge on HBV among health workers; for example only 21.4% and 44% of health workers in Iran (2007) and the UK (2003), knew that HBV could be transmitted by NSIs [22, 23]. However, as of 2018 the prevalence of HBV in the general population in the two countries were reportedly very low for example in Iran it was 1.09% and in the UK, 0.016%. The risks of acquiring HBV among health workers in these two countries is comparatively lower to Ugandan situation where the prevalence of HBV is relatively higher at 17.6%. This may perhaps imply that risks of acquiring HBV among health workers in Uganda may be comparatively higher than the two countries mentioned above. Similarly, two studies on knowledge, attitude and practices (KAP) on HBV infection among health workers in Nigeria in 2009 and 2015 showed that they were generally inadequate [24, 25]. For example in 2011, there was a report that very few studies conducted on health professionals in Nigeria assessed KAP on HBV infection and from the few, most health workers had inadequate knowledge [26]. Health workers in Gulu Regional Referral Hospital had good knowledge on HBV; for example most respondents were aware that hepatitis B vaccination was available and knew the correct dosages.

This high level of knowledge about the presence of HBV vaccine among health workers in Gulu Regional Referral Hospital as revealed by this study could be due to the HBV vaccine's massive sensitization campaigns conducted by the Ugandan Ministry of health in the recent years. This finding is however contrary to the discovery in a South African study conducted in 2008 in Gauteng Province where the majority of health workers 107/161(66.5%) had inadequate knowledge on HBV vaccination [27]. Studies conducted in Nigeria (in 2016) and South Africa (in 2008) reported that health workers who attended infection-prevention training on HBV showed an increased rate of HBV immunization completion than the others [21, 28], findings that are similar to the Pakistani study [28]. Because healthcare workers' knowledge about the HBV vaccine is positively associated with vaccine uptake [21, 28], the Gulu hospital administration could take advantage of the high HBV vaccine awareness among health workers observed by the current study to promote its uptake. Another study conducted in Ethiopia in 2018 showed that basic infection prevention training to all hospital staff is beneficial to the uptake of prevention strategies, increasing health care workers´ compliance to prevention measures to control infectious agents [29]. Training health care workers on infection prevention and control (IPC) could probably improve the uptake of HBV vaccine and other prevention strategies at Gulu Regional Referral hospital and other hospitals where uptake is limited or not available. The study finding that all the five doctor respondents had poor knowledge on the HBV vaccination schedule (Table 1) is worrisome as doctors are supposed to be leaders of the technical teams in hospital, who drive and therefore make final decisions on nearly all clinical decisions. This may have implications on the proper prescription of HBV vaccine and if poor instructions to clients are effected then it would perhaps result into several negative consequences (Table 3). The Expanded Program for Immunization (EPI) program and the Gulu hospital administration will need to organize and administer continuous professional development sessions for all healthcare workers (Table 3, Table 4), particularly doctors, to ensure that HBV vaccination schedules are well understood to improve the HBV vaccine's utilization and effectiveness.

### Attitudes of respondents to hepatitis B virus

Like another review paper published in the journal of Gastroenterology in 2012 [30], the current study found that most respondents had a positive attitude towards HBV vaccination. In particular, the present study found that most health workers were aware that their jobs put them at risk of HBV infection (Figure 1) and this attitude is advantageous for the promotion and uptake of HBV infection prevention measures, including HBV vaccination. In line with another study conducted in Birmingham, UK in 2003, the current study found that most respondents agreed that all patients should be treated as though they had a blood borne pathogen [23]. These positive attitudes towards HBV infection and HBV vaccination among health care workers observed by the current study present an opportunity to the hospital administration and policy makers to introduce HBV vaccination for health care workers in the hospital. The finding that health workers were aware of the dangers posed by HBV infection and the need to maintain protective measures during work to avoid getting infected (Table 5) is advantageous in promoting infection control measures and practices. Positive attitudes towards HBV vaccination among health care workers are positively associated with HBV vaccine uptake [31]. The current study found that students and recent graduate respondents had better attitude towards HBV infection and vaccination than long-serving colleagues and this could indicate the success of the recent drive by the Ugandan Ministry of Education and Sports in incorporating HBV infection and vaccination into the pre-school and medical school curricula. In addition, these authors argue that easy and available information on social and mass media on health related issues especially on infectious diseases and the many incidences of outbreaks in Uganda in the recent years may have in part been the reason for better information on HBV among younger respondents.

**Occupational risks of respondents to hepatitis B virus**

### Pre-exposure risks

In contrast to an Iranian study conducted in 2007 that found that only 27% of health workers said they wore gloves for phlebotomy procedures [22], 69.8% of the respondents in this current study reported high availability and utilization of protective materials for general hygiene and personal protection during contacts with patients. The high utilization of protection gear observed by the current study among the respondents could be due to the training on handling and disposal of infectious material in the hospital most respondents reported to have received in the recent time. The availability of personal protective equipment could also explain their use among the current study respondents. For example, more than half of the respondents reported that gloves were available most of the time and always used them (Figure 2). The finding that more than two-thirds of the current study respondents had high pre-exposure risks to HBV infection (Table 6) is worrisome as it is an indicator that universal infection control precautions are not always being followed, increasing the danger of HBV infection transmission. The finding that more than half of the respondents in the current study did not complete all three doses of HBV as per schedule is similar to those of another study conducted in Northern India in 2013, which found that (62.2%) failed to complete the HBV immunization schedule [7]. The incompletion of HBV vaccination doses by respondents of the current study is worrying as they may not have acquired immunity. Respondents with incomplete HBV vaccination could falsely assume they have acquired the immunity, thus involving themselves in high-risk behavior for HBV infection acquisition and a potential of spreading the virus to the patients they treat and the general population, with devastating consequences.

### Exposure and post-exposure risks of respondents

The current study found that more than half of the respondents had either moderate and high risks to exposure to HBV infection due to the reported work fatigue from long work hours and a lack of regular training on IPC (Table 6). Adhering to universal preventive measures while dealing with blood-related procedures needs to be emphasized as this study found that most respondents (74.4%) were exposed to blood samples of infected HBV patients (Figure 3). The current study found that 23.5% of respondents experienced NSIs is similar to a study conducted in Saudi Arabia in 2002 where the majority of health workers (74%) experienced NSIs [31]. The current and Saudi Arabian studies highlight the low adherence to infection control measures among health workers in many developing countries. The current study found that most (74.1%) of the respondents who got exposed did not get tested for either HBsAg or anti-HBs, indicates a high level of laxity among health workers in following the standard protocols for infection control as stipulated by the Ugandan Ministry of Health guidelines.

Like the current study, only 42.9% of the study respondents completed all three doses of HBV vaccines as per schedule in the Ugandan Ministry of Health guidelines. A study conducted in Nigeria in 2011 too showed poor vaccination status among operating theatre personnel [32]. These avoidable risks need to be addressed as previous studies in many countries have consistently demonstrated low vaccination status among healthcare workers increased their risk of acquiring the HBV infection [33-35]. Healthcare workers are at higher risks of contracting the HBV virus because of their frequent exposures to sharps, blood, and body fluids of infected HBV patients [19, 36, 37]. In a few cases, lack of the necessary personal protection equipments (PPEs) facilitated health workers in breaching infection control protocols during work. The high risk of exposure demands that more preventive approaches must be put in place to ensure that health workers do not acquire the HBV infection, which has far-reaching health consequences to health workers themselves, their patients, and the population. Continuous supervision, availability of IEC materials and training among health care providers on infectious disease control in health care settings could help improve the status of care and awareness.

**Strengths of the study:** the measurements used were adopted from studies drawn from behavioral theories, which are reliable and valid among health workers in many countries. Furthermore, information obtained from this study may contribute to scientific knowledge on HBV among health workers in poor resource settings. In addition, this finding may inform future researches on HBV, a public health challenge among health workers in Africa and many other poor-resourced countries.

**The study limitations:** because of the small sample size and the cross-sectional nature of this study, we could not assess factors responsible for the reported low HBV vaccination coverage among health workers. Secondly, the dependence on self-reported information on HBV vaccination status was another limiting factor. It is not known whether participants would tend to under or over-report their HBV vaccination status. However, the information we obtained reflects the accurate recordings from research participants and may apply to similar settings and environments.

## Conclusion

There is high pre-exposure, moderate-to-high exposure, and post-exposure risks of HBV among health workers in Gulu Regional Referral Hospital. The level of knowledge (good and poor) on HBV infection at almost equal proportion among health workers is awkward. Most health workers knew about the availability of HBV vaccine and the number of doses required to complete the vaccination; however, very few knew the dosing schedules. A substantial number of respondents had not completed their HBV immunization schedule as per the guidelines. However, they reported availability and high utilization of materials for personal protection and hygiene when handling patients in the Hospital. The majority of health workers did not see guidelines and posters on preventing and controlling HBV infections in the Hospital. This might mean that they existed only in few departments or were absent or present, but the majority had not taken note. Regarding exposure and post-exposure experiences, a considerably significant risk exists among health workers in Gulu Hospital. The majority have been exposed (as many as five times in some) or more risky exposure incidents in a few years. There was insufficient exposure and post-exposure management of HBV exposure incidents in the Hospital, with few source patients and exposed health workers tested for HBsAg after an exposure event. The majority of the respondents did not get post-exposure prophylaxis. These reflect the actual dangers to health workers who could potentially acquire HBV infection. Yet, HBV infection among health care workers could easily be avoided by following the Ugandan Ministry of Health guidelines on HBV control and prevention. **Recommendations:** 1) There is a need to reinforce and strengthen awareness strategies for HBV infection, especially on knowledge about the HBV vaccine usage using affordable and effective means such as posters and other IEC materials. Also, more regular staff training on infection control should be put in place. 2) There is a need to maintain, increase the availability and usage of materials for personal protection and general hygiene during contact with all patients in the Hospital. 3) Vaccination policy on HBV should be strengthened and implemented, and follow-up strategies for those vaccinated are put in place to ensure completion of HBV dosing schedule as per guidelines. 4) There is a need to strengthen the proper implementation of exposure and post-exposure management of HBV according to the Ministry of Health (MOH) and WHO guidelines.

### What is known about this topic

- It´s one of the commonest causes of liver complications in Uganda;
- It is highly prevalent in Northern Uganda compared to other parts of Uganda;
- It is endemic in most parts of the sub-Saharan African countries.

### What this study adds

- Health workers in Gulu Hospital have a high pre-exposure and moderate to high exposure and post-exposure risks to Hepatitis B infection;
- Vaccination schedules for Hepatitis B are not completed by most health workers in Gulu Regional Hospital;
- Post exposure management is poor among health workers in Gulu Regional Referral Hospital.

## Annex

**Annex 1**: knowledge, attitude and occupational risks to Hepatitis B infection among health workers in Gulu Regional Referral Hospital, Northern Uganda
